# In Vivo Genotoxicity and Toxicity Assessment of Sterigmatocystin Individually and in Mixture with Aflatoxin B1

**DOI:** 10.3390/toxins15080491

**Published:** 2023-08-03

**Authors:** Maria Alonso-Jauregui, Adela López de Cerain, Amaya Azqueta, Adriana Rodriguez-Garraus, Ana Gloria Gil, Elena González-Peñas, Ariane Vettorazzi

**Affiliations:** 1 MITOX Research Group, Department of Pharmacology and Toxicology, School of Pharmacy and Nutrition, University of Navarra, 31008 Pamplona, Spain; malonso.17@alumni.unav.es (M.A.-J.); acerain@unav.es (A.L.d.C.); amazqueta@unav.es (A.A.); arodriguez.53@alumni.unav.es (A.R.-G.); agil@unav.es (A.G.G.); 2 MITOX Research Group, Department of Pharmaceutical Technology and Chemistry, School of Pharmacy and Nutrition, University of Navarra, 31008 Pamplona, Spain; mgpenas@unav.es

**Keywords:** sterigmatocystin, in vivo, toxicity, genotoxicity, mixture, aflatoxin B1

## Abstract

Mycotoxins are natural food and feed contaminants produced by several molds. The primary mode of exposure in humans and animals is through mixtures. Aflatoxin B1 (AFB1) and sterigmatocystin (STER) are structurally related mycotoxins that share the same biosynthetic route. Few in vivo genotoxicity assays have been performed with STER. In the present genotoxicity study, Wistar rats were dosed orally with STER (20 mg/kg b.w.), AFB1 (0.25 mg/kg b.w.) or a mixture of both in an integrated micronucleus (bone marrow) and comet study (liver and kidney). STER was dosed at the highest feasible dose in corn oil. No increase in the percentage of micronuclei in bone marrow was observed at any condition. Slight DNA damage was detected in the livers of animals treated with AFB1 or the mixture (DNA strand breaks and Fpg (Formamidopyrimidine DNA glycosylase)-sensitive sites, respectively). Plasma, liver, and kidney samples were analyzed with LC-MS/MS demonstrating exposure to both mycotoxins. General toxicity parameters (organs absolute weight, biochemistry, and histopathology) were not altered either individually or in the mixture. The overall absence of individual genotoxicity did not allow us to set any type of interaction in the mixture. However, a possible toxicokinetic interaction was observed.

## 1. Introduction

Mycotoxins are secondary fungal metabolites present as natural contaminants in food and feed, produced mainly by *Aspergillus*, *Penicillium* and *Fusarium* spp. There are more than 300 compounds classified as mycotoxins [1]. Moreover, most of the fungi can produce several mycotoxins, food and feed can be contaminated by several molds, and the complete diet is formed by a diversity of commodities. Therefore, it is probable to find mycotoxins in food and feed, not only individually but in mixtures [2]. In fact, co-occurrence has been widely demonstrated [3,4].

Sterigmatocystin (STER) and aflatoxin B1 (AFB1) are mainly produced by *A. flavus* and *A. parasiticus*. Many other *Aspergillus* species are related to both these species (such as *A. minisclerotigenes*, which is related to *A. flavus*, and *A. novoparasiticus*, which is related to *A. parasiticus*) and are able to produce AFB1 and STER [5]. STER is also produced by *A. nidulans* and *A. versicolor*. This last fungus is the most common source of STER [6]. Both mycotoxins are structurally related and are part of the same biosynthetic route. Indeed, in the biosynthetic pathway, STER is the penultimate precursor before AFB1 and AFG1 [7]. In fact, some studies have demonstrated the co-occurrence of AFB1 and STER in some food products [8,9].

Regarding human exposure, it is widely known that humans are exposed to AFB1. Several human biomonitoring studies have detected AFB1 or AFB1-lysine adducts in blood samples [10]. The last scientific report conducted by the European Food Safety Authority (EFSA) on aflatoxins, confirms the chronic dietary exposure to AFB1 and its association with the risk of hepatocellular carcinoma (HCC) in humans [5]. The evidence of STER human exposure is scarce. Therefore, the risk could not be characterized for human health [6]. A recent assessment conducted by the Joint FAO/WHO Expert Committee on Food Additives (JECFA) also recognized that there are few data on STER occurrence and human exposure [11].

More recent human biomonitoring studies showed the presence of STER in human fluid [11,12,13,14]. However, in some studies STER was only detected after treating the plasma samples with β-glucuronidase/arylsulfatase [12,13]. To the authors knowledge, just one article demonstrates human co-exposure to AFB1 and STER in the same sample [15]. However, considering all the above-mentioned information, human co-exposure to both mycotoxins is expected.

The main health concern for these two mycotoxins is their carcinogenic potential, mainly via a genotoxic mechanism of action. AFB1 is a well-studied mycotoxin with multiple negative effects on humans and animals including inhibition of normal growth, nephrotoxicity, hepatotoxicity and reproductive toxicity, among others. For humans, genotoxicity and carcinogenicity are considered the pivotal health effects [5]. It has been classified as a human carcinogen (group 1) by the International Agency for Research on Cancer (IARC) [16]. To exert its toxicity, an initial bioactivation step is needed. The presence of a double bond at the 8,9-position of the molecule is catalyzed by cytochromes (mainly 3A4, 3A5 and 1A2) into the 8,9-epoxide DNA-reactive form [17]. STER has been classified as possibly carcinogenic to humans (group 2B) [18] and has been recently classified as genotoxic and carcinogenic [11]. STER has shown positive genotoxic responses in bacterial mutations assays [19,20,21] and different cell lines in terms of DNA damage (comet assay and γH2AX), chromosomal aberrations, or micronuclei induction [7,22,23]. Moreover, positive results have been obtained in vivo for chromosomal aberrations [24,25] and DNA damage [26,27]. Two genotoxic modes of action have been proposed. On the one hand, the formation of an epoxy group at the furobenzofuran ring that could bind covalently with DNA [28]. On the other hand, the catechol (catechol 9-hydroxy-STER) formation after the hydroxylation of the aromatic ring has been proposed to contribute to the toxic and genotoxic effects of STER [29].

Recently, both mycotoxins were classified as genotoxic in an in silico—in vitro prioritization strategy applied to 12 mycotoxins. Both were positive in the SOS/umu test after bioactivation with a liver S9 fraction [30]; with kidney S9 fraction, AFB1 was also positive but STER gave equivocal results [31].

STER and AFB1 have been poorly analyzed in binary mixtures in vitro and in vivo [32,33,34,35]. Moreover, there are no genotoxicity studies exploring AFB1 and STER mixtures in vitro or in vivo. Additive proapoptotic effects were observed in HepG2 cells co-exposed to AFB1 and STER binary mixture [34]. However, there were no differences among individual and combined exposure to AFB1 and STER in lipid peroxidation or glutathione redox parameters in carp livers. Contradictory results were obtained in the gene expression analysis; either synergistic or antagonistic effects were hypothesized [35].

The aim of the present study was to analyze, in vivo, the genotoxicity of STER alone and in combination with AFB1. Two genotoxicity assays were combined: (i) the comet assay in its standard version [36] and Fpg (formamidopyrimidine DNA glycosylase)-modified version, that were carried out in liver and kidney tissues and (ii) a micronucleus assay (MN) in bone marrow cells [37]. Moreover, general toxicity parameters such as absolute weight of liver and kidney, clinical biochemistry, and histopathology were also analyzed. Finally, both mycotoxins were quantified in plasma, liver, and kidney with LC-MS/MS with triple quadrupole (QqQ).

## 2. Results

### 2.1. Organ Weight, Clinical Biochemistry and Histopathology

At sacrifice, no differences in liver and kidney absolute weight, organ/body weight index (see Table 1), or biochemical parameters (see Table 2) were found between treated and control groups. Regarding histopathology analysis, AFB1 and STER, individually or in a mixture, did not cause alterations in the liver and kidney (Figures S2 and S3). The histological findings recorded are within the range of normal background levels for rats of this strain and age (Patconsult LAB Study number PAT21033). A higher level of hepatocyte glycogen was observed in animals sacrificed at 24 h that might have been caused by the fasting period.

### 2.2. Standard and Fpg-Modified Alkaline Comet Assay

The results obtained for both versions of the comet assay are shown in Table 3. In the standard version, MMS as PC had a significant increase in the percentage of DNA in the tail compared to NC in both tissues. AFB1 induced a statistically significant increase in the liver (see Table 3). In the kidney, no statistical differences were found in any of the treatments.

In the Fpg-modified version, only the mixture AFB1+STER showed a significant increase in the % of DNA in tail in the liver. Expected results were obtained for AC (59.4% ± 9.4).

### 2.3. Micronucleus Assay

No haematopoietic toxicity was observed in any of the treated groups with the mycotoxins. MMC as PC, showed the highest toxicity (37.3%) (see Table 4).

Considering the genotoxicity, the positive control MMC showed a statistical increase in the number of MN and the percentage of MN. The exposure to AFB1, STER and the mixture did not change none of these two parameters (see Table 4).

### 2.4. Mycotoxin Determination in Plasma, Liver, and Kidney

**Figure 1:**
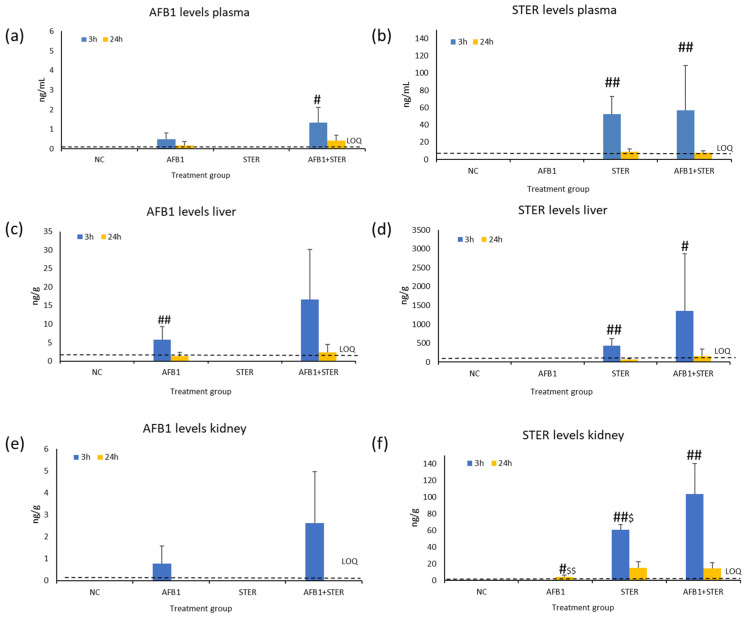
AFB1 quantification of plasma (**a**) (ng/mL), liver (**c**), and kidney (**e**) samples (ng/g) at 3 h and 24 h. STER quantification of plasma (**b**) (ng/mL), liver (**d**), and kidney (**f**) samples (ng/g) for 3 h and 24 h. Dash lines LOQ: limit of quantification. Treatment groups were compared between 3 h and 24 h: (#) significant (*p* < 0.05) and (##) very significant (*p* < 0.01). Additionally, the groups of AFB1 or STER alone were compared to the mixture (3 h and 24 h separately): ($) significant (*p* < 0.05) and ($$) very significant (*p* < 0.01).

Levels of STER and AFB1 in plasma, liver and kidney are shown in Figure 1a–f and in Tables S2 and S5–S11. As expected, none of the mycotoxins were detected in the negative control group. Moreover, no AFB1 levels were detected in the group treated with STER alone. Regarding the group treated with AFB1 alone, no STER levels were detected in the samples, except for the kidney samples, in which levels of STER were found at 24 h in some of the samples. However, all the values were near the LOQ (LOQ: 3.68 ng/g) (see Figure 1f).

Regarding AFB1, higher levels were detected at 3 h than at 24 h in all matrices. This difference was only statistically significant in the liver of the group treated with AFB1 alone and in plasma of the group treated with the mixture. AFB1 levels were consistently higher in the mixture than in the treatment alone in all matrices and at both timepoints. However, there were not statistical differences among these two groups.

In the case of STER levels, again, higher levels were detected at 3 h than at 24 h, with a significant statistical difference in all matrices. In general, more STER was found after the mixture administration than with any of the individual treatments. However, the difference was only statistically significant in the kidney at 3 h.

## 3. Discussion

The present study is a single oral dose genotoxicity study in which STER and AFB1 were administered individually and in a binary mixture. Two genotoxicity endpoints were explored. On the one hand, bone marrow samples were collected for the micronucleus assay. On the other hand, liver and kidney samples were used for the standard and Fpg-modified comet assay. Both are target organs for AFB1 [5] and STER [6] toxicity. These two organs and plasma samples were also used for mycotoxin determination with LC-MS/MS in order to confirm the exposure of the animals to the mycotoxins and to unravel a possible toxicokinetic interaction. The timepoints were 3 h and 24 h. The short treatment (3 h) was used for both versions of the comet assay as it corresponds to the maximum concentration of toxins in plasma and organs. The long timepoint (24 h) was used for the micronucleus test, as the OECD recommends not starting before 24 h after the administration, as it is the time required for cell division and the consequent expression of the micronuclei [37].

The experimental design follows the recommendations of the International Council for Harmonization of Technical Requirements for Pharmaceuticals for Human Use (ICH) [38]: an in vivo assessment of genotoxicity with two different tissues, usually an assay for micronuclei using rodent hematopoietic cells and a second in vivo assay, generally a DNA strand breakage assay, in another tissue. Moreover, following the 3R principle of reduction, replacement, and refinement of animal experiments [39], both tests were combined. The multi-endpoint evaluation with the micronucleus and comet assay in vivo, has been demonstrated to be a suitable design [40] and has been previously used by our group to assess the interaction of AFB1 and OTA in vivo [41].

The doses for both mycotoxins were selected considering previous acute toxicity and genotoxicity studies carried out with both mycotoxins. The acute toxicity of STER is relatively low. However, histopathological changes have been observed at doses lower than the LD50 (120–166 mg/kg b.w.). STER acute toxicity is 10 or more times lower than AFB1 [6]. AFB1 induce acute hepatotoxicity in experimental animals. The LD50 is from 1 to 18 mg/kg b.w. [5]. Moreover, the doses were selected in order to avoid excessive toxicity, as it is known that cytotoxicity can have confounding effects on the comet assay by increasing and/or decreasing DNA [36,42]. For MN assay, it is normally advisable that the highest dose tested do not cause study-limiting toxicity [37].

For AFB1, the dose of 0.25 mg/kg b.w. was selected based in one study from our research group [41], designed to evaluate the interaction between AFB1 and OTA. In this case, the AFB1 dose of 0.25 mg/kg b.w. was dissolved in 0.1 M NaHCO_3_. The solvent selection was based on the good solubility of the other mycotoxin evaluated in the mixture (OTA). In Corcuera et al. (2015), the single oral dose of AFB1 was genotoxic with the standard and modified comet assay (in the liver at 3 h and 24 h) and with the micronucleus assay (at 24 h) in male Fisher F344 rats [41]. Moreover, biochemical and histopathological data revealed mild acute liver damage after 24 h in the groups treated with AFB1 but not at 3 h.

Regarding STER, the dose of 20 mg/kg b.w. was selected based on the study from Dubravka et al. (2020) where three different single oral doses of STER (10, 20, 40 mg/kg b.w.) were tested in male Wistar rats [26]. In this case, the mycotoxin was dissolved in corn oil. All doses were considered positive with the standard and modified comet assay. For our study, we tried to dissolve the highest concentration of 40 mg/kg b.w. (4 mg/mL of corn oil) and it was not soluble. Subsequently, the concentration selected was 20 mg/kg b.w., which was a suspension with turbidity (see Figure S1). For a better preparation of the mixture, corn oil was selected as the solvent of for mycotoxins.

To our knowledge, no in vivo MN assay has been performed to date with STER. Negative results have been obtained in this study but no bone marrow toxicity could be achieved at the highest feasible dose in corn oil. STER exposure could be demonstrated due to the fact of having quantified the mycotoxin in the liver (431 ± 185 ng/g), kidney (61 ± 6 ng/g), and plasma (52 ± 20 ng/mL) after 3 h, and that the mycotoxin could be quantified 24 h after the administration in all the tissues (see Tables S7, S9 and S11). Nevertheless, bone marrow exposure cannot be completely assured. On the other hand, biochemical analysis and histopathology of target organs did not reveal any adverse effect. Thus, it can be concluded that STER did not induce MN in bone marrow PCE production at the highest dose feasible in corn oil.

With respect to the comet assay, after 3 h STER administration, a slight increase in the mean percentage of tail DNA was observed in liver (0.4 vs. 0.8) and kidney (0.4 vs. 1.9), and in the net sensitive sites in both tissues (0.5 vs. 1.5 and 1.2, respectively), but differences were not statistically significant. It should be noted that the OECD guideline for the in vivo comet assay [36], considers that a percentage of DNA in the tail below 6% in rat liver is a good reference for negative controls. This result is not completely in agreement with Dubravka et al. (2020) data interpretation. In this study, liver, and kidney after 24 h of STER administration (10, 20, and 40 mg/kg in corn oil) to Wistar rats were collected. According to the authors, STER had a positive genotoxic response at all the doses tested in both organs, but higher in liver in the standard and hOOG1-modified comet assay. However, it should be noted that in Dubravka et al. (2020) the tail intensity obtained in organs from treated animals, when applying the standard comet assay, was very low, although statistically significant when compared to the negative control (i.e., tail intensities in liver samples around 0.8% in controls and 2.5% in STER-treated animals). Moreover, in the modified comet assay with hOOG1, if the tail intensity obtained with buffer F is subtracted from the hOOG1 incubated samples, as it should be performed for a correct interpretation [43], the response in the STER treated animals is even lower than the obtained with the control animals.

However, it should be noted that the mathematical analysis of the results was different, and data were transformed before the statistical analysis. Regarding the results of the Fpg-modified version, the comparison between Drubravka et al. (2020) and our study is difficult, as in Dubravka et al. (2020) in the damage of the buffer F was high. This is important for interpreting comet assay results. The buffer F is used for dissolving the enzyme for the comet modified version. If the treatment with the buffer F alone exerts damage, the response of the enzyme would be overestimated. Moreover, in our case, we were not able to solubilize STER at the highest dose used Dubravkva et al. (2020).

Regarding AFB1, surprisingly, in the present study, except for a slight response in liver with the comet assay, genotoxicity was not detected in the groups treated with AFB1 (alone or in combination). The main difference between this study and Corcuera et al. (2015) is the rat strain (F344 versus Wistar) and the vehicle used to dissolve the mycotoxins (NaHCO_3_ versus corn oil). Thus, an influence of the vehicle used in the AFB1 toxicokinetic behaviour could be derived. In Corcuera et al. (2015), the AFB1 levels at 3 h were 0.8 ± 0.1 ng/mL in plasma, 8.9 ± 3.3 ng/g in liver and 0.1 ± 0.03 ng/g in kidney; they were around 1.5× fold higher than in the present study. This fact supports the hypothesis of a slightly different kinetic behaviour of AFB1 based on the vehicles and/or rat strains used in both studies and could explain the lower genotoxic response observed in the present study. Other genotoxicity studies carried out with AFB1 used an intraperitoneal route [44] or much higher doses than in the present study [45,46,47,48,49,50,51], and they are not comparable. 

Unfortunately, the lack of a clear genotoxic response obtained with the individual toxins prevent us to discuss in detail the possible interaction between both mycotoxins. In terms of general toxicity and genotoxicity, our results indicate that the mixture does not increase the toxic response compared to the individual exposure.

However, it is very important to point that the plasma and tissue levels of both mycotoxins were always slightly higher in the animals treated with the mixture than in animals treated with the individual toxins. This difference is particularly evident (around 3× fold higher) in the liver and kidney for AFB1 and in the liver for STER (see Supplementary Tables S1–S11). As only the primary compound has been analyzed, a higher level of each mycotoxin may imply less metabolism, indicating a possible toxicokinetic interaction between both mycotoxins. It is known that the toxic effect of AFB1 is attributable to its AFB1-exo-8,9-epoxide reactive metabolite generated after a monooxygenase reaction mainly by cytochromes P450 [5]. On the other hand, although with much less scientific evidence, STER seems to bioactivate also into a reactive epoxide and detoxify mainly via glucuronidation [6]. Apart from the epoxide bioactivation, the hypothesis of the catechol formation and the subsequent adduct formation with thiols could explain STER genotoxicity [29]. The larger amount quantified in the mixture of both mycotoxins could be due to the absence of bioactivation of AFB1 and STER. In the case of the AFB1 and OTA binary mixture, a genotoxic antagonic effect was observed in vitro [52] and in vivo [41]. The hypothesis was that OTA would persist longer in the organism and would block AFB1 bioactivation due to a competition of both mycotoxins towards the same cytochromes [41]. In our case, the joint presence of AFB1 and STER in the mixture would result also in a type of competition for the metabolizing enzymes of the toxins which would block their bioactivation against each other. However, the present study is a preliminary assessment of this mixture and more evidence of the joint toxicokinetics is required for supporting this hypothesis.

## 4. Conclusions

STER is an emerging mycotoxin with some genotoxicity red flags in the literature. There is very little evidence of its in vivo toxicity potential. The present work presents the toxicity and genotoxicity potential of STER individually and in a binary mixture with AFB1, which is a type 1 human carcinogen and is part of the same biosynthetic route than STER. The animals treated with the highest feasible STER dose in corn oil (20 mg/kg b.w.) via an oral route did not present any sign of toxicity, and negative results were obtained in both genotoxicity assays. After 3 h of treatment, STER exposure was demonstrated in the plasma, livers, and kidneys of all the animals. The binary mixture led to higher concentrations of both mycotoxins than the individual treatment. This could result from a toxicokinetic interaction, but further assessment is needed in order to demonstrate this conclusion.

## 5. Materials and Methods

### 5.1. Chemicals and Reagents

Methylmethanesulfonate (MMS) (Ref No. 129925), mitomycin C (MMC) (Ref No. M4287), AFB1 (Ref No. A6636), corn oil, fetal bovine serum (FBS), sodium chloride (NaCl), sodium hydroxide (NaOH), potassium chloride (KCl), sucrose, dimethylsulfoxide (DMSO), agarose (low melting and normal melting point), triton X-100, Trizma-BASE, HEPES, Na_2_EDTA, potassium bromate (KBrO_3_), Bovine Serum Albumin (BSA), DAPI (4′,6-diamino-2-fenilindol), giemsa, DPX slide mounting medium, Whatman filter paper (grade 1), immersion oil, Hartman’s Fixative (Davidson’s Fixative), eosin, acetic acid, acetonitrile (ACN) (gradient grade for liquid chromatography), β-glucuronidase/arylsulfatase enzyme mixture, ammonium formate (≥99.9% trace metal basis), and mycotoxin standards for analytical determination: AFB1 (Ref No. 3409), STER (Ref No. 32986), were obtained from Merck (Darmstadt, Germany). Physiologic serum and isoflurane (IsoVet^®^) were obtained from B. Braun (Melsungen, Germany). STER (Ref nº CAY-11441) was obtained from Cayman Chemical (Ann Arbor, MI, USA). Formaldehyde 4%, paraffin (M.P. 55–58°), hematoxiline from Harris and sodium acetate were obtained from Panreac Applichem (Barcelona, Spain). Ethanol 70° and 99° were obtained from OPPAC (Navarra, Spain). Xylene (isomers mixture) was obtained from VWR chemicals (Radnor, PA, USA), K3E Vacutainer tubes were purchased at Becton Dickinson (Franklin Lakes, NJ, USA). Fpg was obtained from NorGenoTech (Oslo, Norway). RPMI-1640 medium containing d-glucose, l-glutamine, sodium bicarbonate and sodium pyruvate were purchased at Gibco-Thermo Fisher Scientific (Waltham, MA, USA). Additionally, from Gibco, Hanks Ca^2+^ Mg^2+^ without phenol red, phosphate buffer (PBS) without Ca^2+^ Mg^2+^. Penicillin-streptomycin was obtained from Lonza (Basilea, Switzerland). Methanol (LC-MS grade) was obtained from Scharlab (Barcelona, Spain). Formic acid was obtained from Honeywell fluka (Charlotte, NC, MA, USA). For the clinical biochemistry analysis, the albumin (ALB), aspartate aminotransferase (AST), alanine aminotransferase (ALT), creatinine (CREA), total protein, urea, precinorm 1, precinorm 2, calibrator, ISE low standard, ISE high standard, ISE reference solution, ISE diluent, ISE internal standard, ECO-D, NaOH, NaCl, Limpcub2, Limpcub1 were purchased from Sysmex (Kobe, Japan). Deionized water (H_2_O type II, >18 MΩcm resistivity; and H_2_O type I) was obtained from an Ultramatic Type I system from Wasserlab (Navarra, Spain).

### 5.2. Mycotoxins Safety Precautions

AFB1 and STER for animal administration were purchased in powder and afterwards dissolved in a chemical cabinet. During the procedure, the researcher wore a protective mask type FFP3 and double nitrile gloves. These cautionary measures were also carried out by the manipulators who administered the rats or the staff from the animal facilities who cleaned the animal-housing cages. Additionally, for analytical stock solutions preparation. The necropsies were performed with protective mask type FFP3.

### 5.3. Animals

In vivo experiments were approved by the Ethics Committee on Animal Experimentation of the University of Navarra (CEEA Nº 032-21; date of approval: 7 April 2021). Fifty male Wistar rats (8 weeks old) were purchased from (ENVIGO). The rats were randomly distributed into animal-housing cages and weighed the day of the arrival. Weight variation did not exceed ±20%. The acclimation period was 5 days. The environmental conditions were 12 h day/night cycle, temperature 22 °C, relative humidity 55 ± 20%, standard diet, and water ad libitum.

### 5.4. Study Design and Treatments

#### 5.4.1. Dose Selection

AFB1 and STER doses were selected based on Corcuera et al. (2015) and Dubravka et al. (2020), respectively.

#### 5.4.2. In Vivo Study Design

The in vivo comet and MN assays were carried out following the OECD TG 489 and 474, respectively [36,37] (OECD 2016a; OECD 2016b); the standard and Fpg-modified versions of the comet assay were performed. Both in vivo assays were combined in the same short acute design, but with two different timepoints: 3 h for the comet assay, and 24 h for the MN assay. A total of 50 male Wistar rats (average 241 g: min 193 g–max 289 g) were used and distributed in the following treatment groups (single oral dose): corn oil (negative control), AFB1 (0.25 mg/kg b.w.), STER (20 mg/kg b.w.), AFB1+STER (0.25 mg/kg b.w. + 20 mg/kg b.w.) (n = 5 animals per timepoint and treatment). An overnight fasting period was observed prior to the single oral administration by oral gavage. Then, 3 h after the administration, the animals kept for 24 h were fed again. For the positive controls, a separate fifth group (n = 5 animals) was included. For the positive control group, the same animals (n = 5) received an intraperitoneal administration of 4 mg/kg b.w. of MMC (dissolved in physiologic serum) 24 h before being sacrificed and 200 mg/kg b.w. of MMS (dissolved in physiologic serum) via oral gavage 3 h before sacrifice. A reserve group (n = 5 animals) was also included.

### 5.5. Sample Collection

After 3 h or 24 h treatment, the animals were anesthetized for blood extraction and then sacrificed via asphyxia in a CO_2_ cabin. Isoflurane was used as anesthetic and the blood was collected from the retro-orbital plexus in K3E Vacutainer tubes. The blood samples were centrifuged (624× *g*, 10 min, room temperature) to obtain the plasma, which was stored at −80 °C. Plasma was used for biochemical analysis and mycotoxin quantification.

After collection, liver and kidneys were weighed. Right kidneys were analyzed in this study, while left kidneys were stored at −80 °C for future studies. Then, liver and the right kidney were cut into three different pieces for (i) comet assay, (ii) mycotoxin quantification, and (iii) histopathology examination. In all cases, kidney pieces contained both cortex and medulla.

For the comet assay, samples from animals treated for 3 h were used. These pieces were dipped in cold solution (Mg^2+^, Ca^2+^ and phenol red free Hank’s solution containing 20 mM Na_2_EDTA and adjusted to pH 7.5 and supplemented with 10% DMSO just prior to use). Afterwards, tissues were cut into one smaller piece (sections of approximately 2 mm × 2 mm × 2 mm and 2 mm × 3 mm × 5 mm for liver and kidney, respectively). Again, the piece from kidney contained both cortex and medulla. All tissues were used fresh for the comet assay.

For mycotoxins quantification and histopathology, samples from animals treated at 3 h and 24 h were used. The tissue pieces for mycotoxin quantification (LC-MS/MS) were cleaned by blotting them on filter paper and cut into 4 smaller pieces. Afterwards, these 4 pieces were weighed. The average pieces weight for liver was 0.65 g (min: 0.34 g max: 1.12 g). For kidney, all pieces included cortex and medulla and the average piece weight was 0.50 g (min: 0.30 g max: 0.69 g). All pieces were flash-frozen in liquid N_2_ before analysis. Finally, the pieces for histopathology examination were cut (longitudinal cut of one lobule of the liver and transversal cut of the kidney containing cortex and medulla) and fixed in 4% formaldehyde solution.

For MN assay, bone marrows were extracted from the animals treated for 24 h. For that purpose, one of the femurs was obtained and sectioned through trochanters and epicondyles, then centrifuged (827× *g*, 5 min, room temperature). Once the bone marrow was obtained, the extensions were prepared.

### 5.6. Body Weight, Organ/Body Weight Index, Clinical Biochemistry, and Histopathology

Animals were weighed on the day of arrival and the day of the administration. In the necropsy, the organs were weighed just after their extraction. Organ/body weight index was calculated using the following formula: organ weight (g)/body weight measured previously to the administration (g) × 100. 

Biochemical analysis of plasma samples was performed using the biochemical autoanalyzer Cobas c-311. The following parameters were analyzed: total protein (g/dL), albumin (g/dL), urea (mg/dL), aspartate transaminase (AST) (U/L), alanine transaminase (ALT) (U/L), and creatinine (mg/dL).

For the histopathological analysis liver and kidney sections were fixed (as explained in Section 5.5), trimmed, dehydrated, and embedded in paraffin. Sections from the paraffin block were cut at a nominal thickness of 2–4 µm using a microtome and mounted onto glass slides. Finally, the slides were stained with hematoxylin and eosin and cover slipped. Then, a complete microscopic evaluation of the preparations was conducted by PatConsult (Barcelona, Spain).

#### Results Evaluation

The mean and standard deviation (SD) of the results for each group was obtained for the following parameters: body weight, organs absolute weight, and biochemical analysis. The individual data of each animal were compared with the historical data of the laboratory.

### 5.7. Standard and Fpg-Modified Comet Assay

The standard version of the comet assay was performed following the OECD TG 489 [36] (OECD 2016). All samples were kept dipped in the cold solution (see Section 5.5) in ice until processed. Single-cell suspensions were prepared from fresh liver and kidney pieces (see Section 5.5). To do so, one piece was put in a Petri dish on ice and cut extensively in all directions with a scalpel. The resulting cell suspension was suspended in 1.5 mL of new cold solution.

For preparing the gels, 30 µL of the cell suspension was mixed with 140 µL of 1% low-melting-point agarose. Two drops of 70 µL of this mixture were placed on a slide pre-coated with 1% of normal-melting-point agarose. A cover slip (20 mm × 20 mm) was put on top of each drop and gels were allowed to set for 5 min at 4 °C. Three different slides were prepared per sample and labelled as (1) lysis, (2) buffer, and (3) Fpg. Once the gels were solidified, the cover slips were removed and slides were dipped in lysis solution (2.5 M NaCl, 0.1 M Na_2_EDTA, 0.01 M Trizma-BASE, pH 10.5, TRITON X-100 1%, DMSO 10%) at 4 °C for 1–2 h.

For the Fpg-modified comet assay, the slides labelled as ‘buffer’ and ‘Fpg’ were removed from the lysis solution and washed 3 times (5 min each time) with the enzyme reaction buffer (Buffer F: 0.04 M HEPES, 0.1 M KCl, 0.0005 M Na_2_EDTA, 0.2 mg BSA, pH 8) at 4 °C. Then, the samples were treated with 45 µL/gel of Buffer F for the “buffer” slides and Fpg (previously titrated by [53] (Muruzabal et al. 2019)) for the “Fpg” slides and covered with a cover slip (22 mm× 22 mm). “Buffer” and “Fpg” slides were incubated for 1 h at 37 °C in a humid chamber. After the incubation, the cover slips were removed. Thereafter, all the samples (i.e., ‘lysis’-until now in the lysis solution-, ‘buffer’ and ‘Fpg’ slides) were subjected to a high-pH solution (0.3 M NaOH, 0.001 M Na_2_EDTA) for 20 min at 4 °C before an electrophoresis was carried out at 1 V/cm for 20 min in the same solution (4 °C). Finally, all slides were washed in PBS for 10 min and in H_2_O type II for another 10 min before letting the gels to dry at room temperature.

Nucleoids were stained by adding 30 µL of 1 µg/mL DAPI on each gel and placing a cover slip (24 mm × 60 mm) on top. Comets were analyzed in a fluorescence microscopy. A total of 150 comets were scored per slide (75 per gel) using the Comet Assay IV software Instem Perceptive Instruments (Conshohocken, PA, USA). The percentage of DNA in tail was used to describe the comets.

As assay control (AC) for the Fpg-modified comet assay, frozen TK6 cells treated with KBrO_3_ were included in each assay. The AC were obtained by treating TK6 cells at a density of 1 × 10^6^ cells/mL with 2.5 mM KBrO_3_ for 3 h. After treatment, cells were washed via centrifugation, suspended in cell growth medium containing 5% DMSO, aliquoted, and frozen at −80 °C. The aliquots were stored at −80 °C until the assay was carried out (no more than 2 months).

#### Results Evaluation

In each slide, the medians of the % DNA in tail of the 75 comets scored per gel were calculated. Then, the mean of both medians was calculated. In the standard version of the comet assay (“lysis” slides), the % DNA in tail refers to the level of strand breaks (SBs) and alkali labile sites (ALS) present in the sample. For the Fpg-modified version, the net Fpg-sensitive sites were calculated by the subtraction of the % DNA in tail of the slides treated with Buffer F (“buffer” slides) from the slides treated with Fpg (“Fpg” slides).

Mean and standard deviation of each group of treatment were obtained. MIRCA recommendations were followed in these studies. The acceptability criteria established by the OECD guideline TG 489 were checked for the standard comet assay. The criteria for considering a positive outcome were statistical differences (see Section 5.10) between the groups treated with mycotoxins and the negative control in any of the studied parameters. In the case of positive or equivocal results, the histological results from liver and kidney samples are considered, in order to discard the presence of toxicity as false positive.

### 5.8. Micronucleus Assay

The micronucleus assay was performed following the OECD TG 474 [37] (OECD 2016). The procedure was as follows. Ten minutes after the extension preparation (see Section 5.5), the slides were fixed by introducing them in absolute methanol for 10 min. Once fixed, the extensions were introduced in Giemsa 10% in PBS, previously filtered with a Whatman grade 1 filter paper, in low agitation for 10 min. The dye was then removed by gently renewing the content of the bucket with tap water for 2–3 min. Finally, the extensions were introduced in a bucket with type II water for 2 min. Then, the samples were dried up on a Whatman filter upside down for 10 s and face up during 15 min. Finally, samples were analyzed by eye with an optical microscope, with the 100× objective, using immersion oil. Polychromatic erythrocytes (PCE), normochromic erythrocytes (NCE), and MN were scored.

#### Results Evaluation

To evaluate the haematopoietic toxicity, the PCE rate with respect to the total erythrocytes (PCE+NCE) was calculated in a minimum of 500 NCE and was PCE counted. The mean and SD of the PCE rate of the 5 animals composing each group were obtained. To evaluate the genotoxicity, the MN formation was assessed in at least 4000 PCE and the MN % was calculated for each animal by the application of the following formula:

$$\mathrm{MN}\%=\frac{\mathrm{Number}\;\mathrm{of}\;\mathrm{MN}}{\mathrm{total}\;\mathrm{PCE}}\times 100$$

Then, the mean MN % of the 5 animals from each group and their corresponding SDs were obtained. The statistical analysis (Section 5.10) was performed for the PCE rates and % MN. For results interpretation, the acceptability criteria established by the OECD guideline TG 474 for negative and positive controls and adequate number of cells counted were checked. The criteria for considering a positive outcome were statistical differences between the treated groups and the NC in the % of MN.

### 5.9. Determination of Mycotoxins in Plasma, Kidney, and Liver

The concentration of mycotoxins in plasma, liver, and kidney was determined with LC-MS/MS (LC system 1200 series coupled to a 6410 Triple Quadrupole (QqQ) both from Agilent Technologies (Waldbronn, Germany). The analytical method used was that described in [54] with some modifications for sample treatment in order to adjust the procedure to a lower volume of plasma and two new matrices (kidney and liver). Briefly, a chromatographic column Ascentis Express C18, 2.7 µm × 2.1 mm (Supelco Analytical, St. Louis, MO, USA) at 45 °C, and a mobile phase composed of (A) 5 mM ammonium formate with 0.1% formic acid in water, and (B) 5 mM ammonium formate with 0.1% formic acid in 95:5 methanol/water, in gradient conditions and at 0.4 mL/min, were used. Detection was in ESI+ mode and the selected transitions for AFB1 were: 313.0–128.1 (quantification: Q) and 313.0–285.1 (qualification: q); and for STER 325.0–310.1 (Q) and 325.0–281.0 (q).

#### 5.9.1. Sample Preparation

##### Plasma Samples

The enzyme mixture (β-glucuronidase/arylsulfatase) was added to each plasma sample (1:6). After 1 min of vortexing, the samples were incubated overnight at 37 °C in water bath. The next day, plasma was added (100 µL) to a Captiva EMR-lipid cartridge (1 mL) from Agilent Technologies (Santa Clara, CA, USA) in a 1:5 proportion with ACN (with formic acid at 1%) for 5 min and 200 µL of the effluent were evaporated until dry (60 °C) under vacuum. Before chromatographic analysis, the sample was reconstituted with 100 µL of 40% B-mobile phase via vortexing (2 min).

##### Liver and Kidney Samples

Frozen pieces of kidney and liver (see Section 5.5) were homogenized for 2 min in a universal polypropylene sterile container from Deltalab (Barcelona, Spain) with 4 µL of cold sodium phosphate buffer (0.05 M, pH 6.5) per mg of tissue in a polytron PT-MR-3000 homogenizer with a metal rod PT-DA 3012/2 TS Kinematic (Switzerland). After homogenizing each sample, the metal rode was cleaned twice with type II water and once with ethanol. After ethanol evaporation, the rod was cleaned once with the sodium cold buffer and the next sample was put afterwards. The samples were aliquoted (2 aliquots of 100 µL and the universal container with the rest of the homogenate) and stored at −80 °C for at least one day until mycotoxin extraction. All the procedure was performed under cold conditions using ice.

The enzyme mixture (β-glucuronidase/arylsulfatase) was added to each liver or kidney homogenate sample (1:6). After 5 min of vortex, the samples were incubated overnight at 37 °C in water bath. The next day, ACN (with formic acid at 1%) was added to the microtubes with the sample in a 1:5 proportion. Afterwards, the samples were vortexed for 5 min and centrifugated for 5 min at 88× *g*. A volume of 0.4 mL was added to a Captiva EMR-lipid cartridge (1 mL). A vacuum was applied and 200 µL of the effluent were evaporated until dry (60 °C) under vacuum. Thereafter, the sample was reconstituted with 100 µL of 40% B-mobile phase via vortexing (2 min).

#### 5.9.2. Preparation of Calibration Samples

Commercial stocks of AFB1 (2 µg/mL) and STER (50 µg/mL) were diluted 1/8 and 1/50 in ACN, respectively. Then, a stock solution in ACN containing both mycotoxins was prepared. The final concentration of each mycotoxin in the stock solution was 9.2 ng/mL for AFB1 and 45.9 ng/mL for STER. The stock solution was stored at −20°. Prior to use, the stock solution was left at room temperature for 30 min. In order to prepare calibrators, adequate volume (10–300 µL) of the mixed stock solution was poured into 1.5 mL microtubes and dried in an evaporator under vacuum at 60 °C. Then, 5 µL of ACN was added and vortexed for 1 min. This volume was diluted with plasma (500 µL) or liver or kidney homogenates from blank rats (500 µL). Then, the microtubes were vortexed for 1 (plasma) or 5 min (liver and kidney homogenates). Afterwards, calibration samples were processed as indicated in Section 5.9.1.

#### 5.9.3. Results Evaluation

Solutions obtained from each type of samples after treatment were analyzed in batches in which matrix-matched calibrators, prepared as indicated previously, were included. For each analytical batch and mycotoxin, a calibration curve was obtained and used for quantification. Results were accepted if the obtained curve had a minimum of six levels of calibration and if the calculated mycotoxin concentration for each calibrator did not differ from the nominal value by more than 15% [55]. In all the curves, the weighting factor of 1/x was employed. In Table S5 equations obtained for the calibration curves, range and number of calibrators used for each one of them are shown. Moreover, for unequivocal identification of the mycotoxin presence in the sample, both transitions should be present with a q/Q (%) ratio similar (relative error (RE) < 20%) in samples and in calibrators. Additionally, the retention time obtained should be similar in samples and calibrators (RE < 2.5%) [56]. For STER, some samples had concentration levels out of the calibration curve range. In these cases, samples were re-analyzed after diluting the original samples using plasma, liver or kidney homogenates as diluents. After quantification, the mean and standard deviation of each group of treatment was obtained for mycotoxin levels in plasma, liver, and kidney at each study time. For the mean calculation the values < LOQ were considered as 0.

### 5.10. Statistical Analysis

Statistical significance was set up at *p* < 0.05. Very significant results were set up at *p* < 0.01. The inferential study was performed for each parameter through Kruskal–Wallis test. If statistically significant differences were found, the results of each group were subjected to statistical assessment using the non-parametric Mann–Whitney U test comparing each group with the negative control: (*) significant (*p* < 0.05) and (**) very significant (*p* < 0.01). In the case of the standard and Fpg-comet assay and MN assay, the positive control (and AC for Fpg-comet assay) was also compared with the negative control. For mycotoxin quantification in all matrices, the statistical comparisons of the 3 h and 24 h groups in each treatment were ((#) significant (*p* < 0.05) and (##) very significant (*p* < 0.01)), respectively, and the comparisons of the treatment alone of AFB1 or STER and the mixture (($) were significant (*p* < 0.05) and ($$) very significant (*p* < 0.01)), respectively. Statistical analyses were conducted using Stata/IC 12.1.

## Figures and Tables

**Table 1:** Organ absolute weights and organ/body weight indexes. Group mean and standard deviation are shown. The normality ranges of each parameter (bold letter) were calculated from the historical data of the laboratory (n = 69 animals).

Group	Organs Absolute Weight (g)		Organ/Body Weight Index	
	Liver	Kidney	Liver	Kidney
IC 95% (inf-sup)	**7.6–13.5**	**0.7–1.5**	**-**	**-**
NC 3 h	8.4 ± 0.6	0.9 ± 0.07	3.14 ± 0.2	0.4 ± 0.03
AFB1 3 h	7.8 ± 0.7	0.9 ± 0.06	3 ± 0.1	0.3 ± 0.03
STER 3 h	8.9 ± 0.4	0.9 ± 0.07	3.3 ± 0.3	0.3 ± 0.01
AFB1+STER 3 h	7.8 ± 1.2	0.9 ± 0.09	2.9 ± 0.4	0.4 ± 0.02
NC 24 h	11.7 ± 1.1	0.8 ± 0.06	4.1 ± 0.2	0.3 ± 0.03
AFB1 24 h	11.9 ± 2	1.03 ± 0.08	4.1 ± 0.6	0.3 ± 0.02
STER 24 h	12.8 ± 1.1	1.1 ± 0.1	4.2 ± 0.4	0.4 ± 0.03
AFB1+STER 24 h	10.7 ± 2.1	0.9 ± 0.1	3.6 ± 0.6	0.3 ± 0.04

AFB1: aflatoxin B1. NC: negative control. STER: sterigmatocystin. No significant differences were observed with Kruskal–Wallis test. Organ/body weight index has been calculated using the following formula: organ weight (g)/body weight measured previously to the administration (g) × 100.

**Table 2:** Biochemical parameters. Group mean and standard deviation are shown. The normality ranges of each parameter (bold letter) were calculated from the historical data of the laboratory (n = 296 animals).

Group	Total Protein (g/dL)	Albumin (g/dL)	Urea (mg/dL)	AST (U/L)	ALT (U/L)	Creatinine (mg/dL)
IC 95% (inf-sup)	**5.1–6.7**	**3.7–4.7**	**21–58**	**52–138**	**14–63**	**0.17–0.5**
NC 3 h (n = 4)	5.2 ± 0.2	3.5 ± 0.2	27.5 ± 2.4	144 ± 43.6	41 ± 5.2	0.3 ± 0.03
NC 24 h (n = 5)	4.8 ± 0.7	3.2 ± 0.5	20.8 ± 2.2	124.8 ± 50.1	43.8 ± 11.2	0.2 ± 0.03
AFB1 3 h (n = 3)	5.1 ± 0.3	3.5 ± 0.1	26.3 ± 6.6	98 ± 19.7	35 ± 3.6	0.3 ± 0.06
AFB1 24 h (n = 5)	4.9 ± 0.2	3.4 ± 0.2	23.6 ± 2.5	96.4 ± 20.1	41.8 ± 12	0.3 ± 0.03
STER 3 h (n = 4)	5.3 ± 0.6	3.5 ± 0.3	29.7 ± 11.1	131.7 ± 38.4	43.7 ± 5.7	0.2 ± 0.06
STER 24 h (n = 5)	5 ± 0.4	3.3 ± 0.4	20.8 ± 2.3	123 ± 68.4	38.4 ± 8.4	0.2 ± 0.04
AFB1+STER 3 h (n = 4)	5.6 ± 0.4	3.7 ± 0.2	32.7 ± 7.6	121.2 ± 18.6	42.5 ± 7.8	0.3 ± 0.04
AFB1+STER 24 h (n = 3)	4.8 ± 0.5	3.3 ± 0.1	23.3 ± 3.2	107 ± 25.9	48.7 ± 4.7	0.2 ± 0.06

AFB1: aflatoxin B1. ALT: alanine transaminase. AST: aspartate transaminase. NC: negative control. STER: sterigmatocystin. The number of animals is indicated in each group (n). The biochemical analysis was not performed for all animals due to the low volume of sample obtained when the blood was extracted though the retro-orbital plexus. No significant differences were observed with Kruskal–Wallis test.

**Table 3:** Results of the standard and Fpg-modified comet assays carried out in liver and kidney of rats after 3 h of receiving AFB1 (0.25 mg/kg b.w.), STER (20 mg/kg b.w.) or AFBA1+STER in a single oral dose. The table collects the group mean (n = 5) and standard deviation for each parameter evaluated.

Group	Standard (% DNA in Tail)		Fpg-Modified (Fpg-Sensitive Sites)	
	Liver	Kidney	Liver	Kidney
NC	0.4 ± 0.9	0.4 ± 0.3	0.5 ± 0.8	0.5 ± 0.8
AFB1	1.8 ± 1.06 *	1.5 ± 0.9	0.18 ± 0.4	4.4 ± 7.6
STER	0.8 ± 1.2	1.9 ± 1.3	1.5 ± 2	1.2 ± 1.4
AFB1+STER	1.9 ± 1.7	0.3 ± 0.4	2.2 ± 1.6 *	2.03 ± 1.7
PC	17.3 ± 6.3 **	36.4 ± 14.3 *	-	-
AC	-	-	59.4 ± 9.4 **	59.4 ± 9.4 **

AC: Assay control; AFB1: aflatoxin B1. NC: negative control. PC: positive control. STER: sterigmatocystin. The statistical analysis was performed for each parameter through Kruskal–Wallis test. If the result was significant, Mann–Whitney U test was performed comparing each group of treatment with the negative control: (*) significant (*p* < 0.05) and (**) very significant (*p* < 0.01).

**Table 4:** Erythrocyte MN test results in bone marrow samples after 24 h of receiving AFB1 (0.25 mg/kg b.w.), STER (20 mg/kg b.w.) or AFB1+STER in a single oral dose. The table collects the group mean and SD for each parameter evaluated. For individual data check Table S1 (Supplementary Materails).

Group ID	PCE ^1^	NCE	PCE (%)	PCE ^2^	MN	MN (%)
NC	255.2 ± 39.6	266.6 ± 42.4	49 ± 7.8	4003.4 ± 2.9	14 ± 5.3	0.3 ± 0.2
AFB1	242.8 ± 100.4	274.2 ± 108.1	47.2 ± 20.1	4002.4 ± 2.6	20 ± 10.9	0.5 ± 0.3
STER	247.2 ± 34.1	277.2 ± 37.5	47.2 ± 6.7	4003 ± 2	10 ± 2.9	0.2 ± 0.1
AFB1+STER	221.4 ± 83.7	282 ± 89.8	44.7 ± 17.2	4001.8 ± 1.3	11 ± 2.3	0.3 ± 0.04
PC	194.2 ± 86.1	332.2 ± 101	37.3 ± 17.6	4004.8 ± 1.3	36 ± 20.3 *	0.9 ± 0.5 *

AFB1: aflatoxin B1. MN: micronuclei. NC: negative control. NCE: normochromic erythrocytes from a total of 500 erythrocytes approximately (PCE+NCE). PCE ^1^: polychromatic erythrocytes from a total of 500 erythrocytes approximately (PCE+NCE). PCE ^2^: total number of polychromatic erythrocytes analysed. PC: positive control. STER: sterigmatocystin. The statistical study was performed for each parameter through Kruskal–Wallis test. If the result was significant, Mann–Whitney U test was performed comparing each group of treatment with the negative control: (*) significant (*p* < 0.05).

## Data Availability

Data is contained within the article.

## Supplementary Materials

The following supporting information can be downloaded at: https://www.mdpi.com/article/10.3390/toxins15080491/s1, Table S1. Erythrocyte MN test results in bone marrow samples after 24 h of receiving AFB1 (0.25 mg/kg b.w.), STER (20 mg/kg b.w.) or AFB1+STER in a single oral dose. The table collects the group raw data and mean and SD for each parameter evaluated. Table S2: Results of the mycotoxin determination in plasma, liver, and kidney of the single-dose oral genotoxicity study. The table collects the group mean and SD; Table S3: Mean values for retention times (tR) and transitions ratios (q/Q) of the calibrators and the samples for AFB1 in plasma, liver, and kidney, along with their respective relative error (RE) (%) values; Table S4: Mean values for retention times (tR) and transitions ratios (q/Q) of the calibrators and the samples for STER in plasma, liver, and kidney, along with their respective relative error (RE) (%) values; Table S5: Calibration curve equations and descriptors for AFB1 and STER in plasma, liver and kidney; Table S6: AFB1 individual levels in plasma samples (ng/mL) 3 h and 24 h after administration; Table S7: STER individual levels in plasma samples (ng/mL) 3 h and 24 h after administration; Table S8: AFB1 individual levels in liver samples (ng/g) 3 h and 24 h after administration; Table S9: STER individual levels in liver samples (ng/g) 3 h and 24 h after administration; Table S10: AFB1 individual levels in kidney samples (ng/g) 3 h and 24 h after administration; Table S11: STER individual levels in kidney samples (ng/g) from animals administered for 3 h and 24 h; Figure S1: Suspension with turbidity of STER at 2 mg/mL (20 mg/kg b.w.) in corn oil; Figure S2: Microphotographs of the livers (left) and kidneys (right) from the rats treated for 3 h. All images were taken at 10× except for the AFB1 liver (40×). In the bottom of the image there are observations of the status of the organs. NC: negative control. AFB1: aflatoxin B1. STER: sterigmatocystin. A+S: AFB1+STER; Figure S3: Microphotographs of the livers (left) and kidneys (right) from the rats treated for 24 h. All images were taken at 10×. In the bottom of the image there are observations of the status of the organs. NC: negative control. AFB1: aflatoxin B1. STER: sterigmatocystin. A+S: AFB1+STER. Glcogen: glucogen.
