# Supplementary material for: In Vivo Genotoxicity and Toxicity Assessment of Sterigmatocystin Individually and in Mixture with Aflatoxin B1
Source: Toxins (Basel). 2023 Aug 3;15(8):491. doi: 10.3390/toxins15080491 (PMC10467059; doi:10.3390/toxins15080491)
Supplement: Supplementary file 1 [file toxins-15-00491-s001.zip › toxins-2450767-supplementary.pdf]

toxins MDPI

Supplementary Materials: In Vivo Genotoxicity and Toxicity Assessment of Sterigmatocystin Individually and in Mixture with Aflatoxin B1

Maria Alonso-Jauregui, Adela López de Cerain, Amaya Azqueta, Adriana Rodriguez-Garraus, Ana Gloria Gil, Elena González-Peñas and Ariane Vettorazzi

Table S1. Erythrocyte MN test results in bone marrow samples after 24h of receiving AFB1 (0.25 mg/kg b.w.), STER (20 mg/kg b.w.) or AFB1+STER in a single oral dose. The table collects the group raw data and mean and SD for each parameter evaluated.

Group ID	PCE[1]	NCE	PCE (%)	PCE[2]	MN	MN (%)
NC	240	275	46.6	4003	10	0.2
	220	291	43.1	4003	10	0.2
	283	275	50.7	4008	13	0.3
	223	299	42.7	4003	23	0.6
	310	193	61.6	4000	14	0.4
	255.2±39.6	266.6±42.4	49±7.8	4003.4±2.9	14±5.3	0.3±0.2
AFB1	287	214	57.3	4007	12	0.3
	209	320	39.5	4002	14	0.3
	285	245	53.8	4001	17	0.4
	86	436	16.5	4001	39	1.0
	347	156	69	4001	18	0.4
	242.8±100.4	274.2±108.1	47.2±20.1	4002.4±2.6	20±10.9	0.5±0.3
STER	260	250	51.0	4006	10	0.2
	212	319	39.9	4001	14	0.3
	231	295	43.9	4004	11	0.3
	233	295	44.1	4002	9	0.2
	300	227	56.9	4002	6	0.1
	247.2±34.1	277.2±37.5	47.2±6.7	4003±2	10±2.9	0.2±0.1
AFB1+STER	323	181	64.1	4003	11	0.3
	160	343	31.8	4002	13	0.3
	274	227	54.7	4001	7	0.2
	114	403	22.1	4003	12	0.3
	263	256	50.7	4000	12	0.3
	221.4±83.7	282±89.8	44.7±17.2	4001.8±1.3	11±2.3	0.3±0.04
PC	261	240	52.1	4005	11	0.3
	291	218	57.2	4004	23	0.6
	168	390	30.1	4003	43	1.1
	179	357	33.4	4006	40	1.0
	72	456	13.6	4006	64	1.6
	194.2±86.1	332.2±101	37.3±17.6	4004.8±1.3	36±20.3*	0.9±0.5*

AFB1: aflatoxin B1. MN: micronuclei. NC: negative control. NCE: normochromic erythrocytes from a total of 500 erythrocytes approximately (PCE+NCE). PCE1: polychromatic erythrocytes from a total of 500 erythrocytes approximately (PCE+NCE). PCE2: total number of polychromatic erythrocytes analysed. PC: positive control. STER: sterigmatocystin. The statistical study was performed for each parameter through Kruskal-Wallis test. If the result was significant, Mann-Whitney U test was performed comparing each group of treatment with the negative control: (*) significant ($p<0.05$).

Table S2. Results of the mycotoxin determination in plasma, liver, and kidney of the single dose oral genotoxicity study. The table collects the group mean and SD.

Mycotoxin quantified	Group	Plasma (ng/mL)		Liver (ng/g)		Kidney (ng/g)	
		3h	24h	3h	24h	3h	24h
AFB1	NC	<LOQ	<LOQ	<LOQ	<LOQ	<LOQ	<LOQ
	AFB1	0.48±0.34	0.16±0.22	5.79±3.53##	1.37±0.93	0.78±0.80	<LOQ
	STER	<LOQ	<LOQ	<LOQ	<LOQ	<LOQ	<LOQ
	A+S	1.34±0.78#	0.43±0.27	16.59±13.61	2.35±2.09	2.62±2.33	<LOQ
STER	NC	<LOQ	<LOQ	<LOQ	<LOQ	<LOQ	<LOQ
	AFB1	<LOQ	<LOQ	<LOQ	<LOQ	<LOQ	4.04±2.5#$^{\$\$}$
	STER	52.54±20.07##	9.13±3.04	431.14±185.3##	61.51±33.1	60.79±6.36##	14.70±7.80
	A+S	57.09±51.43##	7.73±2.25	1359.7±1515.3#	158.4±185.2	103.7±36.4##	14.58±6.40

AFB1: aflatoxin B1. A+S: AFB1+STER. NC: negative control. STER: sterigmatocystin. The inferential study was performed for each parameter through Kruskal-Wallis test. If the result was significant, Mann-Whitney U test was performed comparing the groups among 3h and 24h in each group of treatment: (#) significant ($p<0.05$) and (##) very significant ($p<0.01$). Also, the groups of AFB1 or STER alone were compared to the mixture (3h and 24h separately): ($^{\$}$) significant ($p<0.05$) and ($^{\$\$}$) very significant ($p<0.01$).

Table S3. Mean values for retention times (t_R) and transitions ratios (q/Q) of the calibrators and the samples for AFB1 in plasma, liver and kidney, along with their respective relative error (RE) (%) values.

t_R plasma		RE (%)	t_R liver		RE (%)	t_R kidney		RE (%)
calibrators	samples		calibrators	samples		calibrators	samples	
4.97	4.97	0	5.47	5.45	0.4	5.34	5.30	0.7
**q/Q plasma**		**RE (%)**	**q/Q liver**		**RE (%)**	**q/Q kidney**		**RE (%)**
calibrators	samples		calibrators	Samples		calibrators	samples	
58.7	49.8	15.2	65.89	63.96	2.9	68.82	64.26	6.6

Table S4. Mean values for retention times (t_R) and transitions ratios (q/Q) of the calibrators and the samples for STER in plasma, liver and kidney, along with their respective relative error (RE) (%) values.

t_R plasma		RE (%)	t_R liver		RE (%)	t_R kidney		RE (%)
calibrators	samples		calibrators	samples		calibrators	samples	
16.38	16.4	0,1	17.08	17.03	0.3	16.91	16.85	0.4
**q/Q plasma**		**RE (%)**	**q/Q liver**		**RE (%)**	**q/Q kidney**		**RE (%)**
calibrators	samples		calibrators	samples		calibrators	samples	
94.03	94.47	0.5	99.66	97.66	2.0	104.89	97.8	6.8

Table S5. Calibration curve equations and descriptors for AFB1 and STER in plasma, liver and kidney.

		Equation	Nº calibrators (RE% <15)	Range (ng/mL)
**AFB1**	Plasma	y=479.94x+23.28	8	0.09-1.8
	Liver	y=191.78x-9.10	6	0.18-5.5
	Kidney	y=424.92x-12.32	10	0.18-5.5
**STER**	Plasma	y=637.84x-1425.92	6	2.7-27.52
	Liver	y=52.47x+55.94	6	0.92-27.52
	Kidney	y=205.79x-36.03	7	0.92-27.52

Table S6. AFB1 individual levels in plasma samples (ng/mL) 3h and 24h after administration.

	M1	M2	M3	M4	M5	Average
			**3h**			
NC	<LOQ	0.18	<LOQ	<LOQ	<LOQ	<LOQ
AFB1	0.59	0.15	0.19	1.00	0.47	0.48
STER	<LOQ	0.14	<LOQ	<LOQ	<LOQ	<LOQ
A+S	1.12	2.13	0.87	0.41	2.17	1.34
			**24h**			
NC	<LOQ	<LOQ	<LOQ	<LOQ	<LOQ	<LOQ
AFB1	<LOQ	0.33	<LOQ	<LOQ	0.46	0.16
STER	<LOQ	<LOQ	<LOQ	0.27	<LOQ	<LOQ
A+S	0.79	0.24	0.28	0.19	0.63	0.43

AFB1: aflatoxin B1. A+S: AFB1+STER. M1-5: male rats from 1 to 5. NC: negative control. LOQ: limit of quantification (0.09 ng/mL). STER: sterigmatocystin.

Table S7. STER individual levels in plasma samples (ng/mL) 3h and 24h after administration.

	M1	M2	M3	M4	M5	Average
			**3h**			
NC	<LOQ	<LOQ	<LOQ	<LOQ	<LOQ	<LOQ
AFB1	<LOQ	<LOQ	<LOQ	<LOQ	<LOQ	<LOQ
STER	63.62	50.28	57.39	19.56	71.85	52.54
A+S	121.54	25.88	20.03	13.88	104.10	57.09
			**24h**			
NC	<LOQ	<LOQ	<LOQ	<LOQ	<LOQ	<LOQ
AFB1	<LOQ	2.71	<LOQ	<LOQ	<LOQ	<LOQ
STER	8.96	8.47	8.22	14.13	5.85	9.12
A+S	7.28	6.06	8.87	11.00	5.43	7.73

AFB1: aflatoxin B1. A+S: AFB1+STER. M1-5: male rats from 1 to 5. NC: negative control. LOQ: limit of quantification (2.7 ng/mL). STER: sterigmatocystin.

Table S8. AFB1 individual levels in liver samples (ng/g) 3h and 24h after administration.

	M1	M2	M3	M4	M5	Average
			**3h**			
NC	<LOQ	<LOQ	<LOQ	<LOQ	<LOQ	<LOQ
AFB1	5.87	2.60	6.42	11.28	2.76	5.79
STER	<LOQ	<LOQ	2.81	<LOQ	<LOQ	<LOQ
A+S	19.19	24.00	<LOQ	6.05	33.72	16.59
			**24h**			
NC	<LOQ	<LOQ	<LOQ	<LOQ	<LOQ	<LOQ
AFB1	0.80	2.05	<LOQ	1.79	2.19	1.44
STER	<LOQ	<LOQ	<LOQ	<LOQ	<LOQ	<LOQ
A+S	5.57	1.19	<LOQ	2.20	2.80	2.35

AFB1: aflatoxin B1. A+S: AFB1+STER. M1-5: male rats from 1 to 5. NC: negative control. LOQ: limit of quantification (0.72 ng/g). STER: sterigmatocystin.

Table S9. STER individual levels in liver samples (ng/g) 3h and 24h after administration.

	M1	M2	M3	M4	M5	Average
			**3h**			
NC	<LOQ	<LOQ	5.26	<LOQ	<LOQ	<LOQ
AFB1	3.70	<LOQ	<LOQ	<LOQ	<LOQ	<LOQ
STER	642.91	413.09	165.23	369.96	564.53	431.14
A+S	985.28	478.49	4022.36	349.21	963.38	1359.74
			**24h**			
NC	<LOQ	<LOQ	6.78	10.86	<LOQ	<LOQ
AFB1	<LOQ	<LOQ	8.83	9.31	<LOQ	<LOQ
STER	63.85	112.99	43.19	24.21	63.33	61.51
A+S	45.40	63.33	112.30	486.76	84.36	158.43

AFB1: aflatoxin B1. A+S: AFB1+STER. M1-5: male rats from 1 to 5. NC: negative control. LOQ: limit of quantification (3.68 ng/g). STER: sterigmatocystin.

Table S10. AFB1 individual levels in kidney samples (ng/g) 3h and 24h after administration.

	M1	M2	M3	M4	M5	Average
			**3h**			
NC	<LOQ	<LOQ	<LOQ	<LOQ	<LOQ	<LOQ
AFB1	<LOQ	<LOQ	1.10	1.90	0.89	0.8
STER	<LOQ	<LOQ	1.70	<LOQ	<LOQ	<LOQ
A+S	1.68	5.71	<LOQ	1.40	4.32	2.6
			**24h**			
NC	<LOQ	<LOQ	<LOQ	<LOQ	<LOQ	<LOQ
AFB1	<LOQ	<LOQ	<LOQ	<LOQ	<LOQ	<LOQ
STER	<LOQ	<LOQ	<LOQ	<LOQ	<LOQ	<LOQ
A+S	1.74	<LOQ	<LOQ	<LOQ	<LOQ	<LOQ

AFB1: aflatoxin B1. A+S: AFB1+STER. M1-5: male rats from 1 to 5. NC: negative control. LOQ: limit of quantification (0.72 ng/g). STER: sterigmatocystin.

Table S11. STER individual levels in kidney samples (ng/g) from animals administered for 3h and 24h.

	M1	M2	M3	M4	M5	Average
			**3h**			
NC	<LOQ	<LOQ	4.67	<LOQ	4.02	<LOQ
AFB1	<LOQ	<LOQ	<LOQ	<LOQ	4.13	<LOQ
STER	68.00	65.70	60.41	52.02	57.81	60.79
A+S	106.14	85.84	101.21	61.74	160.40	103.7
			**24h**			
NC	<LOQ	4.10	3.85	5.01	<LOQ	<LOQ
AFB1	<LOQ	4.52	6.78	4.42	4.47	4.04
STER	14.87	27.58	10.54	7.02	13.50	14.70
A+S	9.72	10.88	13.96	25.64	12.72	14.58

AFB1: aflatoxin B1. A+S: AFB1+STER. M1-5: male rats from 1 to 5. NC: negative control. LOQ: limit of quantification (3.68 ng/g). STER: sterigmatocystin.

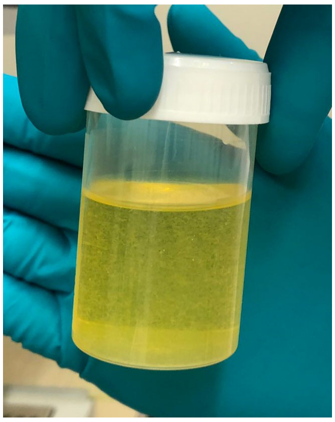

Figure S1. Suspension with turbidity of STER at 2 mg/mL (20 mg/kg b.w.) in corn oil.

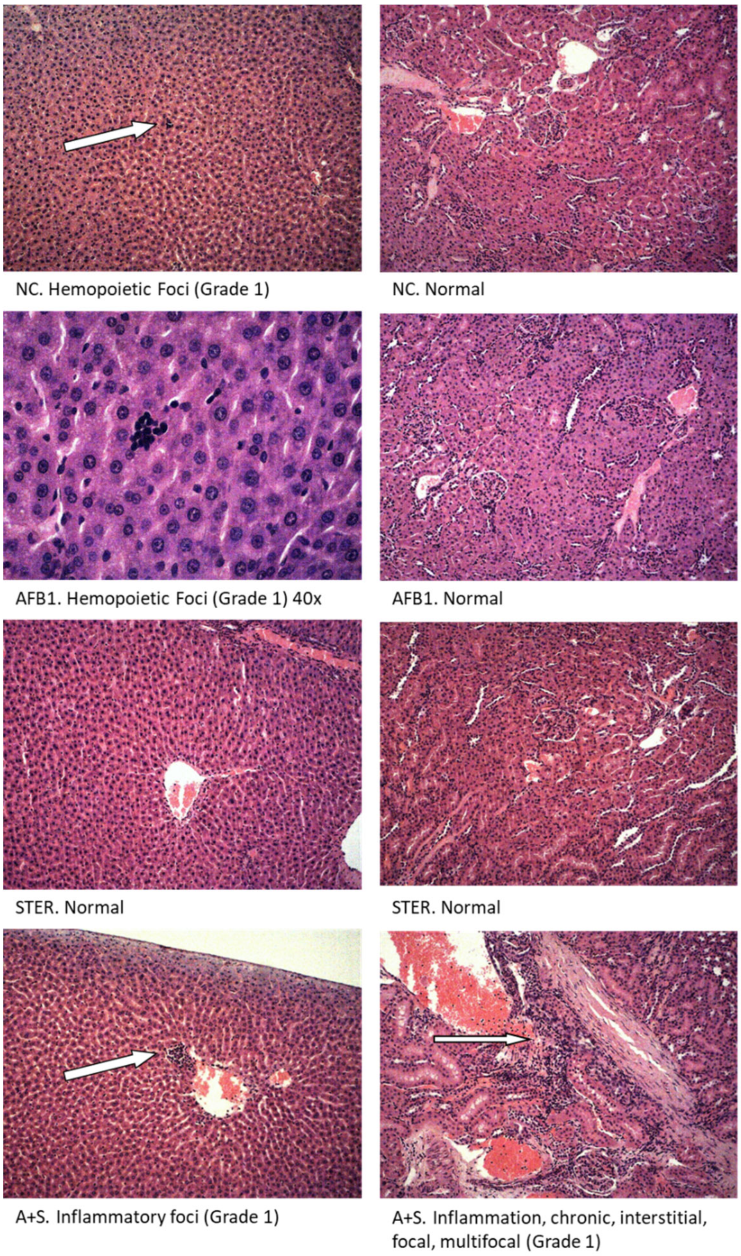

Figure S2. Microphotographs of the livers (left) and kidneys (right) from the rats treated 3h. All images were taken at 10x except for the AFB1 liver (40x). In the bottom of the image there are observations of the status of the organs. NC: negative control. AFB1: aflatoxin B1. STER: sterigmatocystin. A+S: AFB1+STER.

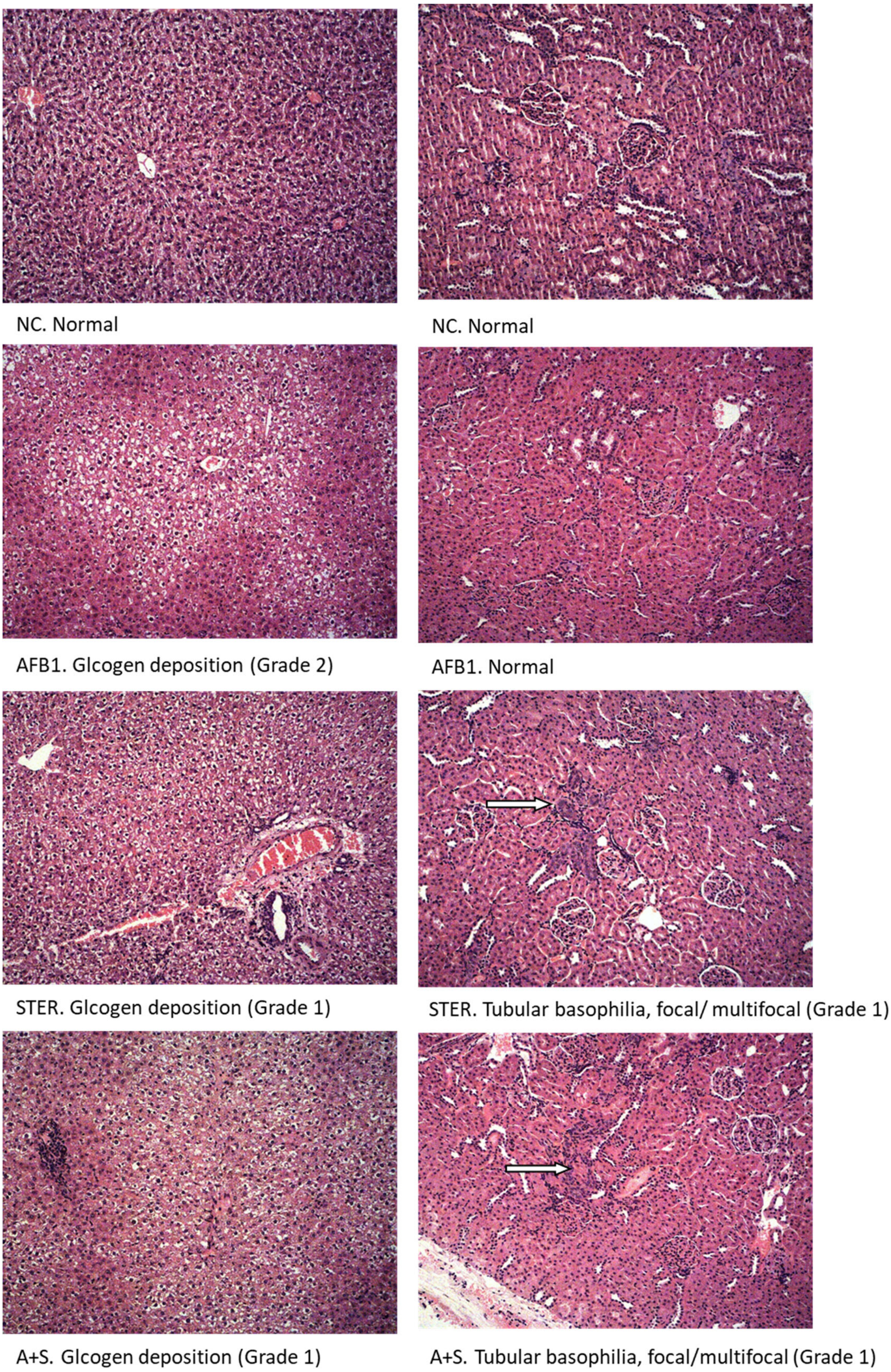

Figure S3. Microphotographs of the livers (left) and kidneys (right) from the rats treated 24h. All images were taken at 10x. In the bottom of the image there are observations of the status of the organs. NC: negative control. AFB1: aflatoxin B1. STER: sterigmatocystin. A+S: AFB1+STER. Glcogen: glucogen.
